# The Pancreatic Islet Regulome Browser

**DOI:** 10.3389/fgene.2017.00013

**Published:** 2017-02-14

**Authors:** Loris Mularoni, Mireia Ramos-Rodríguez, Lorenzo Pasquali

**Affiliations:** ^1^Research Program on Biomedical Informatics, IMIM Hospital del Mar Medical Research Institute and Universitat Pompeu FabraBarcelona, Spain; ^2^Biomedical Genomics, Institute for Research in Biomedicine, The Barcelona Institute of Science and TechnologyBarcelona, Spain; ^3^Program of Predictive and Personalized Medicine of Cancer, Germans Trias i Pujol University Hospital and Research InstituteBadalona, Spain; ^4^Germans Trias i Pujol Campus, Josep Carreras Leukaemia Research InstituteBadalona, Spain; ^5^CIBER de Diabetes y Enfermedades Metabólicas AsociadasBarcelona, Spain

**Keywords:** pancreatic islet, epigenome, non-coding DNA, gene regulation, β-cells

## Abstract

The pancreatic islet is a highly specialized tissue embedded in the exocrine pancreas whose primary function is that of controlling glucose homeostasis. Thus, understanding the transcriptional control of islet-cell may help to puzzle out the pathogenesis of glucose metabolism disorders. Integrative computational analyses of transcriptomic and epigenomic data allows predicting genomic coordinates of putative regulatory elements across the genome and, decipher tissue-specific functions of the non-coding genome. We herein present the Islet Regulome Browser, a tool that allows fast access and exploration of pancreatic islet epigenomic and transcriptomic data produced by different labs worldwide. The Islet Regulome Browser is now accessible on the internet or may be installed locally. It allows uploading custom tracks as well as providing interactive access to a wealth of information including Genome-Wide Association Studies (GWAS) variants, different classes of regulatory elements, together with enhancer clusters, stretch-enhancers and transcription factor binding sites in pancreatic progenitors and adult human pancreatic islets. Integration and visualization of such data may allow a deeper understanding of the regulatory networks driving tissue-specific transcription and guide the identification of regulatory variants. We believe that such tool will facilitate the access to pancreatic islet public genomic datasets providing a major boost to functional genomics studies in glucose metabolism related traits including diabetes.

## Introduction

During the last decade, the advent of high-throughput “-omics” technologies, has greatly promoted advances in the study of human diseases at the genomic, transcriptomic, and epigenomic levels. Sequence databases and software analysis tools are now crucial tools for molecular biologist to understand the molecular mechanisms underlying tissue-specific functions. Nevertheless, the systematic acquisition of large bioinformatic datasets has created a tremendous gap between available data and their biological interpretation. Frameworks to access processed and integrated genomic datasets may assist, computational and non-computational scientists, to bridge this gap and provide understanding and biological interpretations to the regulatory and transcriptional complexity of the genome.

In this context genome browsers are key tools in the accomplishment of this task. The UCSC Genome Browser (Speir et al., 2016), ENSEMBL (Yates et al., 2016) and NCBI's Sequence Viewer (Wolfsberg, 2011), for example, provide to the research community a wealth of integrated information and represent nowadays essential instruments to assist the interpretation of genomic data.

The pancreatic islets of Langerhans constitute an endocrine tissue embedded in the exocrine pancreas and represent the sole source of insulin in the human body. Pancreatic islets play a crucial role in maintaining normal glucose homeostasis, and islet-cell dysfunction and/or reduction in islet-cell mass are key elements in the development of diabetes mellitus. For these reasons, understanding the regulatory networks controlling the tissue-specific expression of pancreatic islets, is key to shed light on the molecular mechanisms underlying diabetes.

Large consortia such as ENCODE (Dunham et al., 2012) and the Epigenome Roadmap (Bernstein et al., 2010) provided extensive epigenetics maps allowing annotation of the non-coding regions of the human genome for a large amount of cell lines and tissues including several relevant to diabetes such as adipose tissue and skeletal muscle, while other less accessible primary tissues such as the endocrine pancreas were not prioritized in these studies. For their central role in diabetes pathogenesis, different laboratories embarked in profiling the transcriptomic and epigenetic landscape of human pancreatic islet-cells (Bhandare et al., 2010; Gaulton et al., 2010; Stitzel et al., 2010; Parker et al., 2013; Dayeh et al., 2014; Pasquali et al., 2014) in an ongoing effort to shed light on the pancreatic islets tissue-specific gene regulation. Free access to such data represents an invaluable opportunity for the research community to dissect the molecular mechanisms of glucose metabolism diseases (Ashcroft and Rorsman, 2012). Nevertheless, these datasets are deposited in different repositories, often in bulky raw format files, thus of difficult immediate access especially to non-bioinformatic users.

Here we present the Islet Regulome Browser, an intuitive web tool providing access to interactive exploration of a wealth of pancreatic islet genomic data allowing the visualization of different classes of regulatory elements and transcription factor binding sites obtained from experiments performed by different labs worldwide. The Islet Regulome Browser is addressed to molecular biologists, human geneticist and clinicians with or without bioinformatics skills.

## Materials and methods

**Figure 1:**
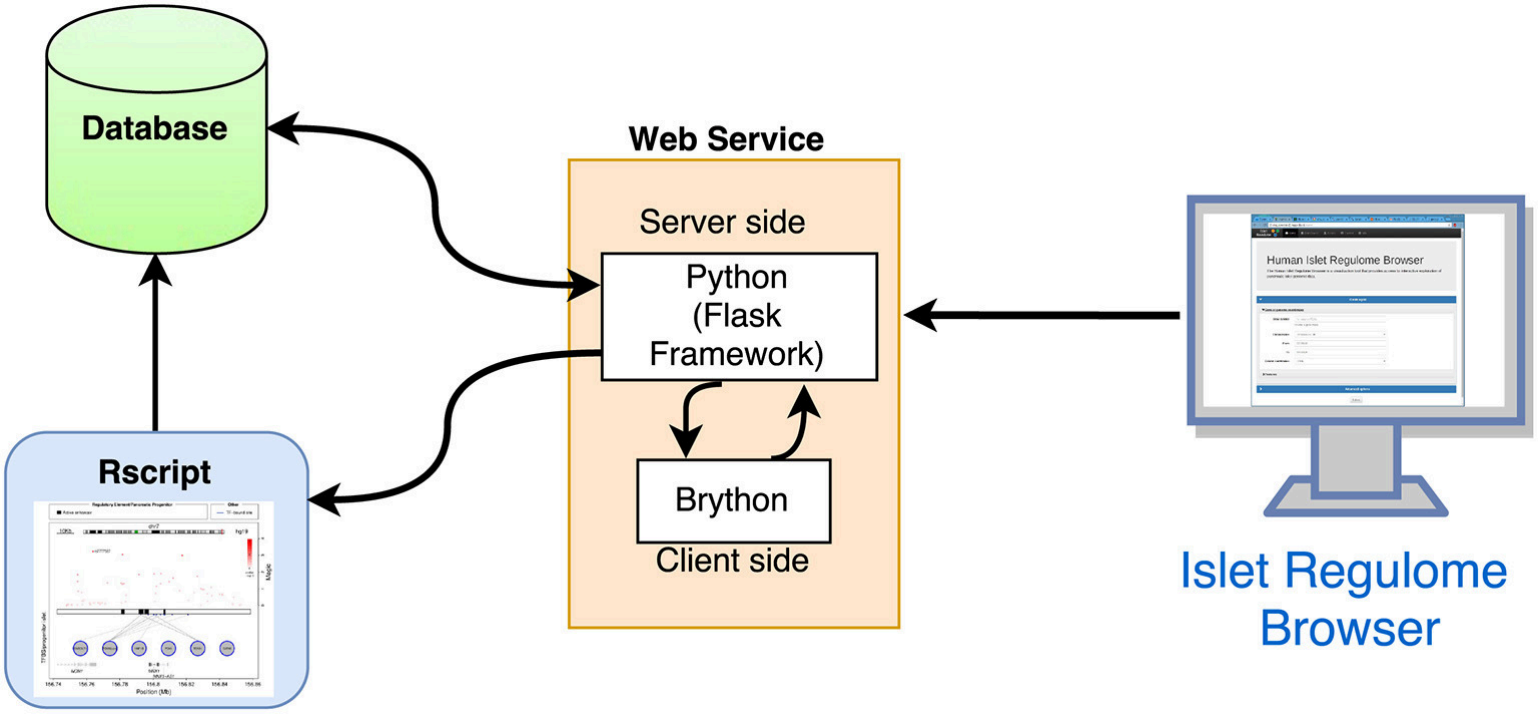
**Structure of the Islet Regulome Browser**. Schematic representation of the interactions between the 3 main components of the Islet Regulome Browser web application.

The overall structure of the Islet Regulome Browser is illustrated in Figure 1.

The Islet Regulome Browser internal structure is composed of three main components: (a) the database, which is saved in binary format as RData objects and tabix indexed files, (b) the code for computing the graphic image, written in R (Rizzo^1^), and (c) the interface and the framework for the web service, written in Python (http://www.python.org). The Islet Regulome Browser is compatible with all the most popular web browsers and operative systems. It can be explored via web at http://www.isletregulome.com or can be installed in a workstation or laptop through the Python package management system with the command *pip install regulome_web*. The source code is available under the MIT license at https://bitbucket.org/batterio/regulome_web.

### Web interface and plot generation

The code for running the Islet Regulome Browser is composed of two main blocks that interact with each other: a Python framework that creates the web interface and retrieves the user input, and the R code which generates the plot and the tables.

On the server side the Islet Regulome Browser is managed by the Flask framework (http://flask.pocoo.org/). The web interface allows users to generate plots and tables by querying for a gene name or for a specific genomic region. In addition, users can customize their analyses by choosing which datasets to use. The interactivity of the web application is achieved by using Brython (http://brython.info/), a Python 3 implementation for client-side web programming. The options selected by the user are forwarded to the R script that generates both the plot and the result tables (Figure 1).

The plot is generated by an R script (R version 3.3.1) that takes as input the user specified features, such as the genomic location and the datasets to use. Several Bioconductor packages have been used to read the database and render the final plot: *Rsamtools* (Morgan et al., 2016), *rtracklayer* (Lawrence et al., 2009), and *Sushi* (Phanstiel et al., 2014). A plot may also be generated via command line, using the code as a stand-alone script.

The plots are converted from PDF to PNG format by the ImageMagick converter tool (http://www.imagemagick.org/) and cached, along with the produced text tables. This allows to rapidly load a plot, instead of generating a new one, in case the same query is repeated. The cache is not used when the users upload their own data.

### Code structure and development

We deposited the Islet Regulome Browser code in a publicly accessible Bitbucket repository (https://bitbucket.org/batterio/regulome_web). Even though the web application can be explored at http://www.isletregulome.com, we created a Python package to easily install the Islet Regulome Browser on a personal computer. The recommended way to install the package is by using the Python package management system (*pip install regulome_web)*. The main requirement for the web application is Python (version 3.5 or above), R (version 3.3.1 and above), and ImageMagik (http://www.imagemagick.org). Other Python related dependencies are listed in the “requirement.txt” file, however, by using the Python package management system all the libraries are automatically installed. Once installed, the Islet Regulome Browser can be executed with the command *regulome_web*. The program has two sub-commands: init and start. *regulome_web init* will create several folders following a structure required by the program, and a configuration file that needs to be modified by the user. The sub-command *regulome_web start* runs the Islet Regulome Browser web server, locally accessible at the url *localhost:5000*.

The R code to render the plot contains two main scripts: (1) *plot_IRB_main.R*, which is the script that needs to be executed to call all other scripts and to draw each part of the plot. (2), *plot_IRB_config.R* contains all configuration variables, including the path to the database. The R script are integrated in the web application but they can also be used via command prompt as a stand-alone program.

### Database

Central to the system is the database, which stores the genomic annotations, chromatin tracks, genome-wide association study (GWAS) variants and transcription factor binding sites that may be visualized by the browser. The publicly available data that can be currently visualized by the Islet Regulome Browser consists of transcription factor binding sites obtained from ChIP-seq experiments in adult human pancreatic islets (PDX1, FOXA2, NKX2.2, NKX6.1, and MAFB) (Pasquali et al., 2014) and pancreatic progenitors (PDX1, FOXA2, ONECUT1, HNF1B, and TEAD1) (Cebola et al., 2015); open chromatin classes and chromatin states in adult pancreatic islets (Parker et al., 2013; Pasquali et al., 2014), enhancer predictions in pancreatic progenitors (Cebola et al., 2015); enhancer clusters and stretched enhancers in adult pancreatic islets (Parker et al., 2013; Pasquali et al., 2014); open chromatin profiles of α- and β-cells FACS purified form adult human pancreatic islets (Ackermann et al., 2016); expression data obtained from RNA-seq experiments including coding (Morán et al., 2012) and non-coding RNA in adult pancreatic islets (Akerman et al., in press), and datasets for genome wide association studies for type 2 diabetes, DIAGRAM (Cho et al., 2012) and fasting glycemia, MAGIC (Scott et al., 2012).

While the above description summarizes the data currently available, the Islet Regulome Browser is a dynamic project. We periodically revise the database and the literature with the aim of providing the most updated and relevant datasets to the pancreatic islet community. We will ensure the future maintenance the Islet Regulome Browser and will interact with other members of the pancreatic islets community to collect their feedback and improve the user interaction with browser.

For each dataset visualized in the browser we provide, in the “*Data Source*” page, full reference of publication as well as links to the repositories where the raw data was deposited for bulk download.

## Results

The Islet Regulome Browser (http://www.isletregulome.com) provides interactive access to a wealth of information, allowing the visualization of GWAS variants, different classes of regulatory elements, together with enhancer clusters, stretch-enhancers and transcription factor binding sites in pancreatic progenitors and adult human pancreatic islets. Integration and visualization of such data may help in the interpretation of the regulatory networks driving tissue-specific transcription and guide the identification of regulatory variants.

**Figure 2:**
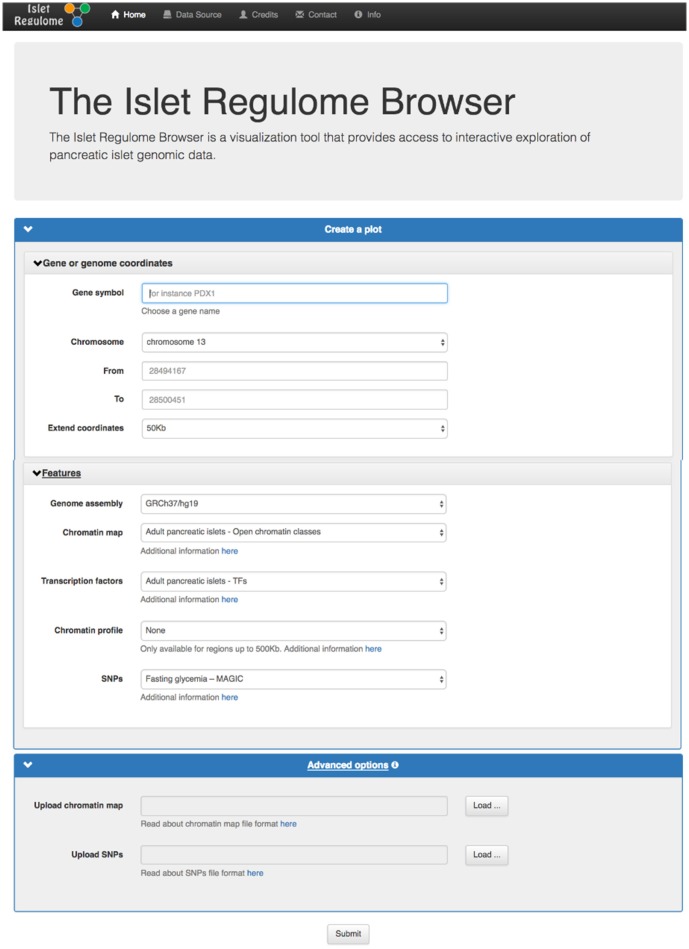
**Front panel of the Islet Regulome Browser**. From the initial page the user can generate a plot by selecting the desired parameters and a valid gene name or an absolute chromosomal location.

From the initial page (Figure 2) a plot can be generated by selecting a valid gene name or an absolute chromosomal location by specifying the genomic coordinates (chromosome, start, and end). The available human builds are: hg18, hg19 (default), and hg38. The plot can be extended at both sides of the gene/location by selecting a range that by default is 50 Kb. To limit the computational load on the server, on the web applications, plots can span a maximum 5 Mb of genomic space and a minimum of 10 bp. These restrictions can be changed in a local installation of the Islet Regulome Browser. Four major track types can be loaded to obtain the desired plot. (1) Tracks named “chromatin maps” refer to genomic maps of regions that may be involved in gene transcription regulation. Such publicly available maps were inferred from experimental datasets such as open chromatin and histone modification profiles, performed in adult human pancreatic islets and pancreatic progenitors. (2) “transcription factors” tracks are maps of transcription factors binding sites obtained from Chip-seq experiments performed in human adult pancreatic islets and pancreatic progenitors. (3) “SNPs” tracks include GWAS variants datasets associated to type 2 diabetes and fasting glycemia. (4) An optional “chromatin profile” track can be loaded to visualize open chromatin profiles obtained from ATAC-Seq experiments performed in FACS purified alpha and beta cells (Figure 2).

Variants or chromatin maps tracks can be uploaded by the user for temporary display from the home page, “*Advanced options*” section. The file size of the uploaded file should not exceed 50 Mb. If a file contains a header, this should start with the “#” symbol. A “*variant file”* should consist of three or four tab-delimited fields. Mandatory fields are those of chromosome, position, and *p*-value. The files can also contain an optional fourth field with the reference number of the variant, additional columns will be ignored. A “*chromatin map file”* has a typical BED file format and should be composed of 3 tab-delimited fields: chromosome, start, and end, additional columns will be ignored. The fields with positional information should only contain integer values while the *p*-values should be numerical values. Upon data upload, a “*Share uploaded files*” option may be selected. This will provide a link that can be copy-pasted to a browser address bar in order to reproduce the Islet Regulome Browser session in use, including the uploaded data. Such link may be shared with other users in order to share data on the Islet Regulome Browser. Data uploaded by the user will be available for 1 month.

### Plot description

**Figure 3:**
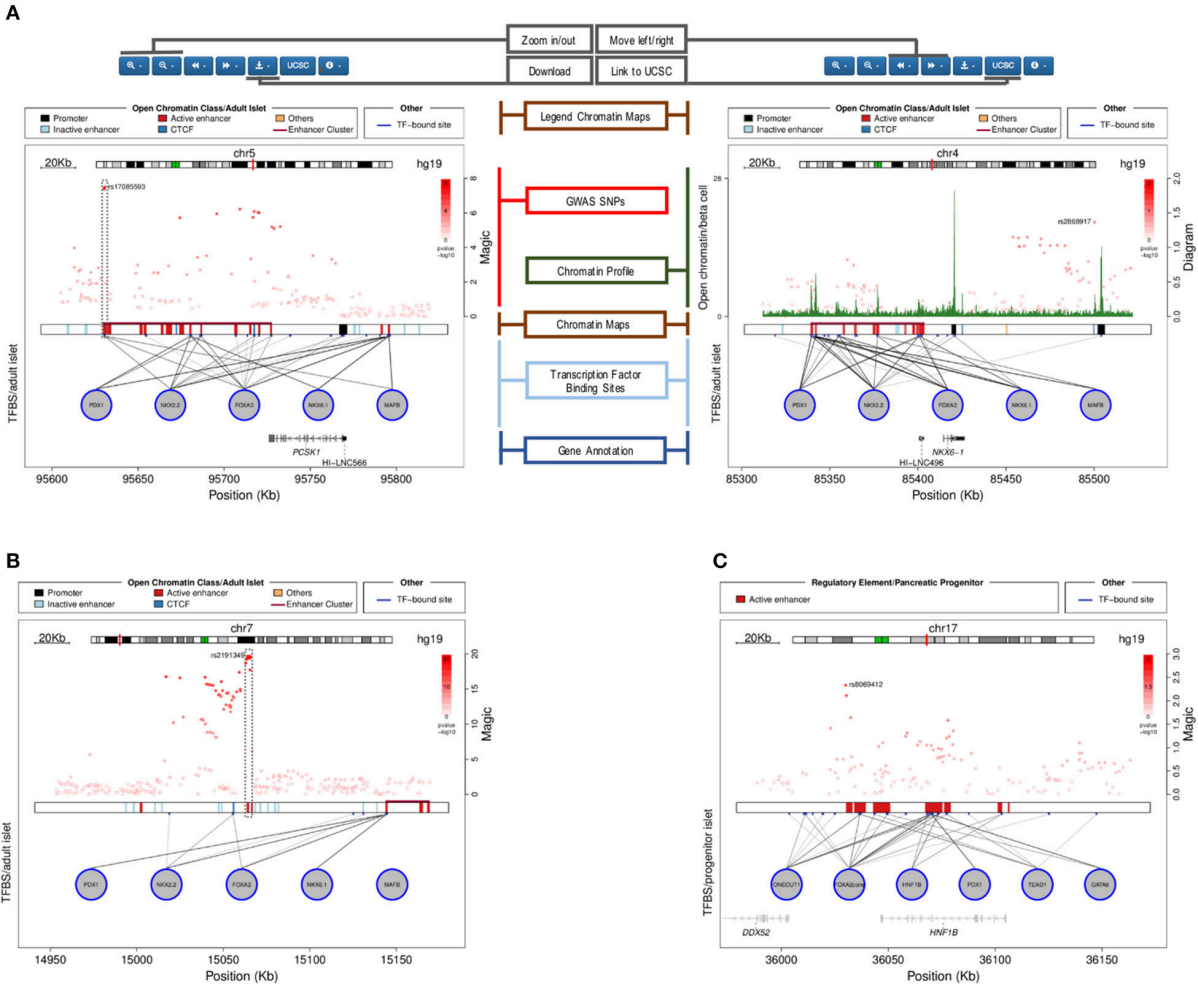
**Plots generated by the Islet Regulome Browser. (A)** Illustration of the different sections of the plot, see the text for the detailed description. The left panel shows an illustrative example of a fasting glycemia associated locus (proximal to the *PCSK1* gene), depicting highly associated SNPs mapping to active regulatory elements (dashed box). The right panel plot illustrates the adult islet regulatory landscape at the locus transcribing the β-cell specific transcription factor NKX6.1. The locus is characterized by a large Enhancer Cluster upstream the gene annotation. **(B)** Example of a fasting glycemia associated locus in proximity of the *DGKB* gene. As for the previous example, the integration of GWAS data with regulatory elements and transcription factors binding sites, allows pinpointing associated variants that directly map to active enhancer sites (dashed box). **(C)** Islet Regulome Browser view of the pancreatic progenitors regulatory landscape at chromosome 17q12. The locus is characterized by a high density of active enhancer elements bound by multiple transcription factors in proximity of the gene encoding HNF1B, a transcription factors involved in pancreatic development and homeostasis.

For any given gene or genomic region selected by the user, a plot is generated (Figure 3).

The plot illustrates the regulatory regions, transcription factors binding sites and GWAS variants in which the sequence of the base genome is represented on the horizontal axis. In the upper part of the plot a red line on the chromosome ideogram reflects the portion of the chromosome displayed.

Each dot represents a genomic variant, being the color intensity of the dot proportional to -Log *p*-value of association, as indicated on the side of the plot. A black box in the central part of the plot contains vertical colored bands depicting different chromatin states, open chromatin classes or regulatory elements as described in the legend above the plot. Black lines connecting the circles (each representing a different transcription factor) to the black box, point to the genomic location of each transcription factor binding site. The color intensity of such lines is proportional to the number of co-bound transcription factors. Annotated genes are depicted as horizontal gray lines at the bottom of the plot, with transcriptional orientation indicated by arrows. Boxes along the line correspond to positions of coding exons. Islet-specific genes are shown in dark gray.

### Plotting versatility

Graphical outputs are highly dynamic, being rendered on the fly. The user can zoom in and out at different resolutions as well as slide left or right 25, 50, and 75% of the length of the plot.

The “*Data displayed*” panel, selectable from top left corner of the plot page, allows reviewing all the settings used to make the plot including genomic coordinates, genome build and all the features selected.

### Retrieve results

**Figure 4:**
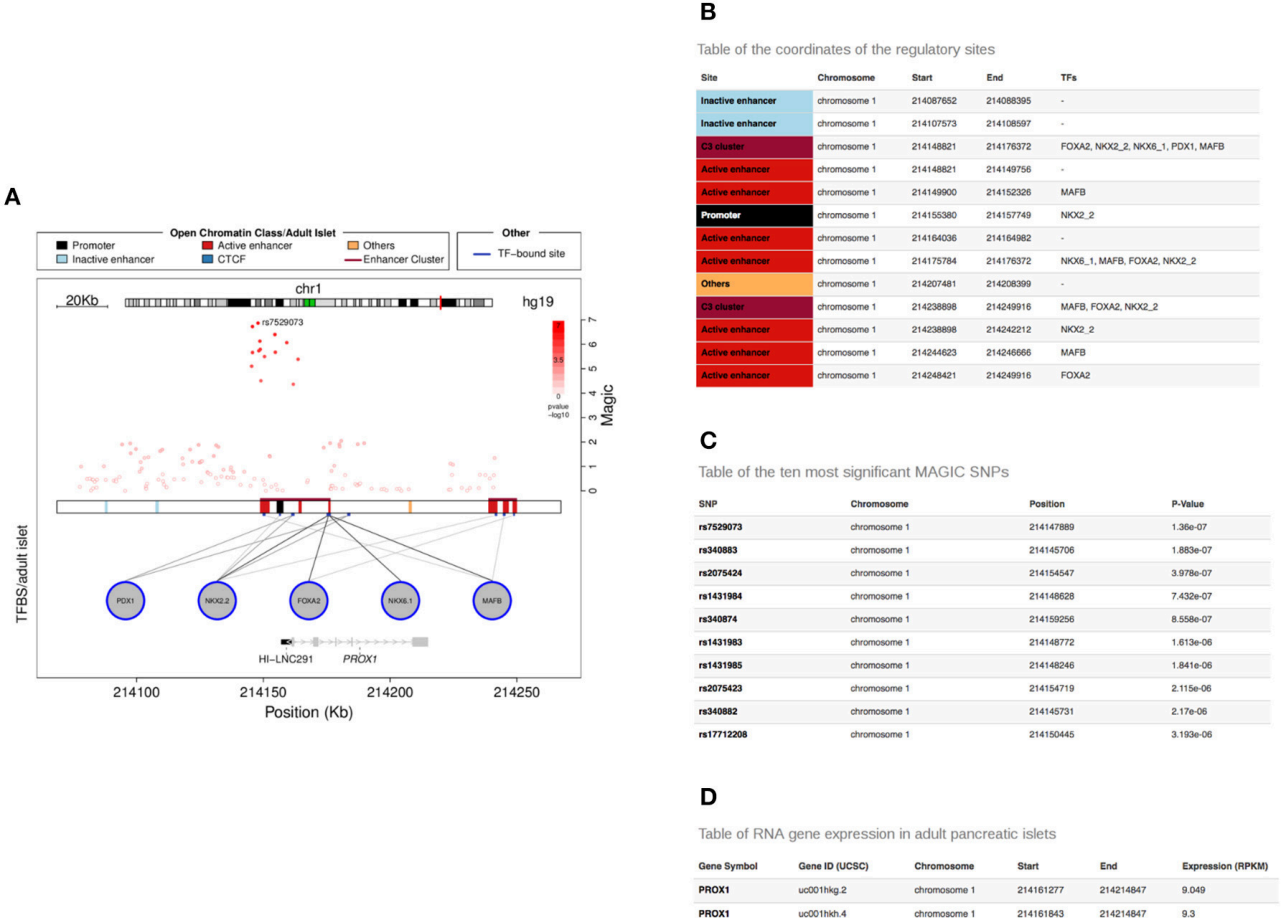
**Tables generated by the Islet Regulome Browser**. Example of tables generated by the Islet Regulome Browser related to the PROX1 gene locus **(A)**. The tables include information on the coordinates of the regulatory elements and transcription factor bindings **(B)**, the GWAS variants along with their *p*-value of association **(C)** and the reference ID and expression level of the different transcript isoforms overlapping the selected locus **(D)**.

Graphical representations and text tables are available for download (Figure 4A).

The plot can be downloaded as PNG (Portable Network Graphics) or as PDF (Adobe Portable Document) format by clicking on the download icon above the plot. The difference between the two formats is that the latter uses vector graphics that is more suitable for high resolution publication figures while PNG compresses the image to a bitmap.

A button above the plot provides a link to a UCSC browser (Speir et al., 2016) session containing all the data currently available in the Islet Regulome Browser for classic UCSC visualization. For this purpose bigwig files were generated from BAM files obtained by aligning the raw data using Bowtie2 (Langmead and Salzberg, 2012) (default parameters).

Three tables related to the selected locus can be downloaded from the “*Table*” panel, selectable from the top left corner of the plot page. One table contains the regulatory regions, open chromatin classes or chromatin states selected for display along with the transcription factors whose binding sites overlap them (Figure 4B). A second table lists the variants contained in the selected locus along with their *p*-value of association (Figure 4C). Finally a third table includes reference ID and expression level of the different transcript isoforms overlapping the selected locus (Figure 4D).

A link at the top left corner of the plot page named “*Data displayed*” redirects the user to the “*Data Source*” used to create the plot displayed, including reference, date of publication and links to the databases where the raw data is deposited.

## Discussion

With the advent of high-throughput sequencing technologies we are assisting to an exponential production of data relevant to different fields of research including pancreatic islet regulatory genomics. Scientists are now facing new challenges by shifting the research efforts from data acquisition to data processing, and knowledge extraction. The role of the Islet Regulome Browser is to provide to the pancreatic islet community fast accessibility to processed genomic data obtained from experiments performed on the endocrine pancreatic tissue. Such data is otherwise of difficult accessibility to non-bioinformatics laboratories being publicly available but usually deposited in bulky unprocessed formats.

Much of the scientific effort in the pancreatic islet field is nowadays dedicated to the understanding of the non-coding genome functions in diabetes, in an effort of translating the GWAS genetic signal of association to a molecular mechanism. Compared to preliminary meeting communications (Ramos et al., 2016) the Islet Regulome Browser now allows the visualization of different classes of regulatory elements and transcription factor binding sites obtained from experiments performed by different labs worldwide. The original view of the data provided by the Islet Regulome Browser allows to easily integrating GWAS raw files with epigenomic and transcriptomic datasets. The user can thus visualize the whole spectrum of variants with different *p*-values of association and contrast them with non-coding regulatory elements and transcription factor binding sites in simple way. We believe that such level of data integration is novel compared to other available genome browsers and can assist researchers in prioritizing diabetes associated variants and to boost their functional validations.

The Islet Regulome Browser is not intended to compete with other genomic browser tools rather to integrate data of specific interest to a relative small scientific community with genomic annotation and epigenetic features obtained from other tissues. To this end we provide the data available at the Islet Regulome Browser processed and organized in UCSC genomic browser sessions as well as direct links to the raw fastq files.

The Islet Regulome Browser is an intuitive interface to explore pancreatic islet genomic datasets. Publicly available experimental data sets such as open chromatin assays, transcription factor binding assays or GWAS variants are readily visualized at loci of interest and provided in the form of summary tables, facilitating the selection of candidate loci to be considered in experimental settings. We believe that such tool will facilitate the access to pancreatic islet public genomic datasets providing a major boost to functional genomics studies in glucose metabolism related traits including diabetes.

The Islet Regulome Browser is freely accessible at http://www.isletregulome.com.

## Author contributions

LP and LM conceived the project. LM designed and implemented the interface, the Web page, and the R code with contribution from MR. LP wrote the paper with contributions from LM and MR. All the authors read and approved it.

## Funding

This work was supported by a grant from the Spanish Ministry of Economy and Competiveness (BFU2014-58150-R). LP is a recipient of a Ramon y Cajal contract from the Spanish Ministry of Economy and Competitiveness (RYC-2013-12864).

### Conflict of interest statement

The authors declare that the research was conducted in the absence of any commercial or financial relationships that could be construed as a potential conflict of interest.

## Footnotes

^1^Statistical computing with R.
